# Integrating GHS into the Ghrelin System

**DOI:** 10.1155/2010/879503

**Published:** 2010-03-18

**Authors:** Johannes D. Veldhuis, Cyril Y. Bowers

**Affiliations:** ^1^Department of Medicine, Endocrine Research Unit, Mayo School of Graduate Medical Education, Clinical Translational Science Center, Mayo Clinic, Rochester, MN 55905, USA; ^2^Division of Endocrinology, Department of Internal Medicine, Tulane University Health Sciences Center, New Orleans, LA 70112, USA

## Abstract

Oligopeptide derivatives of metenkephalin were found to stimulate growth-hormone (GH) release directly by pituitary somatotrope cells in vitro in 1977. Members of this class of peptides and nonpeptidyl mimetics are referred to as GH secretagogues (GHSs). A specific guanosine triphosphatate-binding protein-associated heptahelical transmembrane receptor for GHS was cloned in 1996. An endogenous ligand for the GHS receptor, acylghrelin, was identified in 1999. Expression of ghrelin and homonymous receptor occurs in the brain, pituitary gland, stomach, endothelium/vascular smooth muscle, pancreas, placenta, intestine, heart, bone, and other tissues. Principal actions of this peptidergic system include stimulation of GH release via combined hypothalamopituitary mechanisms, orexigenesis (appetitive enhancement), insulinostasis (inhibition of insulin secretion), cardiovascular effects (decreased mean arterial pressure and vasodilation), stimulation of gastric motility and acid secretion, adipogenesis with repression of fat oxidation, and antiapoptosis (antagonism of endothelial, neuronal, and cardiomyocyte death). The array of known and proposed interactions of ghrelin with key metabolic signals makes ghrelin and its receptor prime targets for drug development.

## 1. Overview

**Figure 1:**
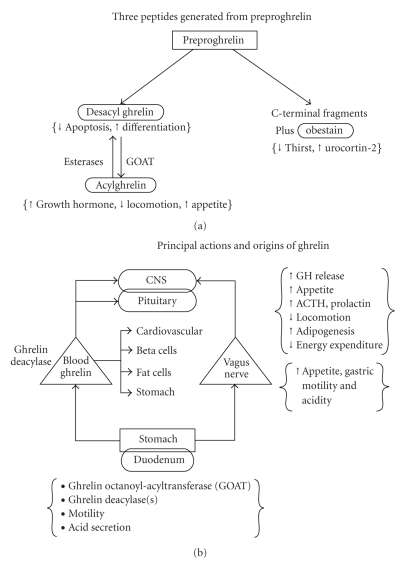
Principal peptide products of preproghrelin (a) and primary actions of ghrelin recognized to date (b) (unpublished line drawing).

 Fundamental questions in peptide biology are the extent to which any given peptide operates in isolation versus interdependently, locally or systemically, and via a single pleiotropic or multiple distinct receptors. Identification of the ghrelin/GHS family initially disclosed GH-releasing properties [1]. Investigations subsequently unveiled multiorgan expression [2–4], multivariate actions [5], and complex modulation of and by collateral effectors [1, 4]: Table 1. The burgeoning repertoire of ghrelin actions mimics that of inhibin and activin [6, 7], which were originally isolated as regulators of follicle-stimulating hormone secretion, and thereafter recognized for hematopoietic and oncologic activity. Analogously, prominent clinical applications of ghrelin/GHS may involve not only GH-stimulating effects but also appetitive, metabolic, cardiovascular, locomotive, and gastrointestinal signaling: Figure 1. Recent development of transgenic mice expressing ghrelin-eGFP (enhanced green fluorescent protein) should permit more detailed mapping of ghrelin-expressing neurons in hypothalamic arcuate and ventromedial nuclei [8–10] and ghrelin-expressing cells in gastric oxyntic glands, pancreatic islets (epsilon cells), the anterior pituitary gland, bone marrow, and other less well-studied sites [4, 11, 12].

## 2. Unique Facets of Ghrelin

### 2.1. Multiplicity of Roles

The etymology of ghrelin is “ghre” for “growth”, consonant with the report by Bowers et al. of direct pituitary GH-releasing effects of metenkephalin-derived synthetic oligopeptides three decades ago [1]. The peptides were initially termed GH-releasing peptides (GHRPs), and more recently GH secretagogues (GHSs) to include congeneric molecules [2]. Multiple peptidyl and several nonpeptidyl analogs of ghrelin (GHS) have been developed and assessed functionally over the last three decades. The ghrelin receptor was cloned in 1996 by Howard et al. based upon phospholipase C-mediated intracellular inositol triphosphatate-dependent Ca^2+^ signal generation in transfected cells [5]. In certain systems, such as pituitary somatotropes, gastric oxyntic cells, appetitive neurons, pancreatic islets, and endothelial cells, additional messengers like phosphoinositidyl-3-kinase, Akt/protein kinase B, adenosine monophosphate protein kinase (AMP kinase), and nitric oxide may modulate ghrelin's actions [13–20]: Table 1. Mitogen-activated protein kinases (MAPKs) and extracellularly regulated kinases (ERKs 1/2) may mediate certain of ghrelin's proliferative and antiapoptic effects [15, 21, 22].

### 2.2. GH-Releasing Mechanisms

**Figure 2:**
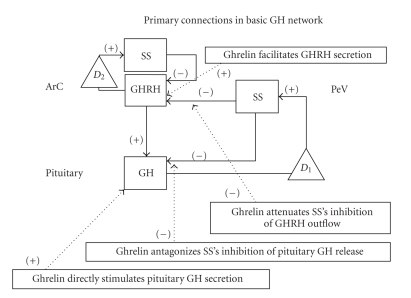
Model-based functional networks subserving GH secretion, showing major effects of GH-releasing hormone (GHRH) and somatostatin (SS) as modified by GHS (ghrelin). *D*_1_ and *D*_2_ denote time delays. ArC and PeV define arcuate and periventricular nuclei (adapted from [41]).

Ghrelin and cognate receptor, GHS-R1a, are distinct entirely from GH-releasing hormone, GHRH, and its receptor isolated in 1982 by the Guillemain and Vale laboratories from human pancreatic neoplasms [23, 24]. The homotypic GHRH receptor is an adenylyl cyclase-activating seven transmembrane-spanning GTP-binding protein [25]. GHRH neurons in the arcuate nucleus express GHS-R1a, but hypothalamic ghrelin neurons do not manifest GHRH receptors [4]. Both neuronal ensembles are extensive and project to the external layer of the pituitary stalk [11]. Nonetheless, to date, any direct role for hypothalamic release of ghrelin to the pituitary gland remains undocumented [26]. Ghrelin is unusual in that existence of the receptor was predicted three decades and cloned three years, before the natural ligand was identified by Kojima et al. in 1999 [3]. The ghrelin receptor exhibits high basal activity even unstimulated. Mutational disruption of constitutive GHS-R1a activity is associated with short stature in humans [27], whereas overexpression of GHS-R1a on GHRH neurons augments postweaning growth, reduces fat mass, and augments GHRH and GH gene expression in mice [28]. Thus, GHS-R1a is an amplifier of GHRH outflow as well as a direct effector of pituitary GH secretion. Exogenous GHS partially overcomes GH inhibition by glucose and infused somatostatin [29, 30]. Neurophysiological data and immediate-early gene responses have revealed two additional mechanisms of ghrelin action, namely, partial antagonism of noncompetitive hypothalamic and pituitary inhibition by somatostatin and of hypothalamic melanocortin and leptin pathways [31–40]. Figure 2 depicts a model, which incorporates several major actions of ghrelin/GHS within the GH axis [41]. This model does not include pituitary ghrelin, which is downregulated by excessive thyroxine, glucocorticoid, or brain-GH feedback and upregulated by GHRH [42]. Selective silencing of the pituitary ghrelin gene will be required to discern its physiological role.

### 2.3. Structural Features

Ghrelin is unique as the first acylated peptide recognized in mammals requiring a short-chain fatty-acid (octanoyl, decanoyl, or decenoyl) moiety linked to the third N-terminal amino acid (usually serine) for primary biological activity, namely. GHRH and GH release, locomotor suppression and appetite stimulation [26, 43–45]. Decanoyl and octanoyl moieties seem equipotent. The enzyme mediating Ser^3^ acylation was cloned by the Brown and Goldstein laboratory in 2008 and named ghrelin octanoyl-acyltransferase (GOAT) [46–48]. GOAT is one of 16 members of membrane-bound O-acyltransferases [49]. It is expressed in pancreas, stomach, skeletal muscle, heart, intestine, bone, and other organs and is endproduct inhibited and fatty-acyl substrate and fasting induced [50–52]. GOAT protein and transcripts exist in individual ghrelin-containing gastric mucosal oxyntic cells [53]. Regulation may exist pretranslationally also, since natural antisense ghrelin RNA's can be demonstrated by analysis of human genomic DNA [54, 55]. A second acyltransferase, termed microsomal zinc-stimulated serine (Ser^2^ ghrelin) octanoyltransferase, distinct from GOAT, was cloned by Ozawa et al. in 2009 [56]. This enzyme is concentrated in the endoplasmic reticulum of human erythroleukemia cells. Its in vivo role is not yet known but might include negative regulation of GOAT via the octanoylated products generated. 

 Unacylated ghrelin competes with acylghrelin for GHS-R1a to a negligible degree, namely, with a *k*_*d*_ of 13 *μ*M, which is four orders of magnitude greater than that for bioactive peptide [57]. Deacylases (esterases) of ghrelin exist in the blood and stomach, which may be nonselective [58, 59]. GOAT can utilize both ghrelin (1–4) and ghrelin (1–5) as substrates for serine acylation, consistent with their core structure [46, 60]. Des-Gln acylghrelin (1–27), an alternative transcript lacking glutamine in N-terminal position 14, is also fully active on the GHS-R1a receptor [61]. Ghrelin's amino-acid sequence is significantly conserved in fish, reptiles, amphibia, birds, and mammals [62]. Congeneric molecules include GHRP-2, GHRP-6, hexarelin, ipamorelin, ibutamorelin, and the nonpeptide MK-0677, which like ghrelin rapidly induce inositol triphosphate, diacylglycerol, and Ca^2+^ release via the GHS-R1a [51, 63–67].

### 2.4. Blood-Borne Ghrelin

**Figure 3:**
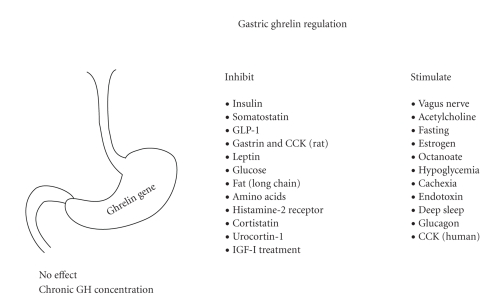
Key hormonal, gastrointestinal, nutritional, stress-related, infectious, and physiological regulators of gastric ghrelin secretion inferred in mammalian species.

Ghrelin concentrations in blood comprise principally desacyl-ghrelin (85%–90%) and in lesser amounts acylghrelin (10%–15%) and C-terminal proghrelin peptides [68, 69]. In large cohorts, ghrelin is higher in women than men [70] and declines with age, body mass index (BMI), hypertension, and other markers of the metabolic syndrome [70]. The inverse relationships between ghrelin and both BMI and insulin concentrations appear to explain much of the age effect [71]. Appropriate sample collection and storage are necessary to limit ghrelin deacylation before assay [72, 73]. Although its exact role is not known, desacyl-ghrelin can exert a variety of agonistic and antagonistic actions [74–76], as discussed further in the relation to the ghrelin receptor. Octanoylated and total ghrelin levels in the stomach and blood rise between meals, during fasting, in cachexia, anorexia, or malnutrition, type I (insulinopenic) diabetes mellitus, after acute endotoxin exposure, overnight during initial deep sleep and in response to estradiol (E_2_), acute hypoglycemia, glucagon infusion, vagal stimulation, and chronic octanoate ingestion [77–89]: Figure 3. In mice, plasma bioactive decanoylated ghrelin increases and octanoylated ghrelin paradoxically decreases during fasting [90], suggesting precise posttranslational control [72]. Unlike gastric ghrelin, hypothalamic ghrelin gene transcript and peptide levels fall during short-term fasting [9]. 

 In various studies, serum total ghrelin concentrations correlate positively with E_2_, IGFBP-1, and creatinine concentrations, and negatively with somatostatin, insulin, thyroxine, leptin, and testosterone (T) concentrations and arterial blood pressure [70, 82, 86, 91–98]. Thus, ghrelin levels rise not only in fasting individuals but also in estrogen/progestin-treated women [99–101], combined antiandrogen and progestin-treated men [82], and the estrogen-rich milieu of the late follicular phase of the menstrual cycle in one but not another study [92, 102]. Conversely, ghrelin levels fall in the high-testosterone milieu of male puberty [93]. Gastric ghrelin-secreting cells express estrogen receptor-alpha [103], thus potentially mediating some sex-steroid effects. Indeed, gonadal downregulation in girls and parenteral (but not transdermal) testosterone administration in boys diminish plasma ghrelin concentrations [104, 105]. In addition, clinical investigations indicate that E_2_ or T supplementation can potentiate GH secretion stimulated by a fixed submaximal dose of GHS/ghrelin [106–112]. On the other hand, low-dose transdermal E_2_ administration did not augment maximal GH stimulation by ghrelin in postmenopausal women [113], and the potentiating action of T administration on GHS was not evident in the dog, rat, or older men [114, 115]. Thus, more data are needed on the developmental dependence of sex-steroidal facilitation of GHS action. The differences before and after puberty do not seem to be due to feedback by differing GH levels, since acute infusion of GH or a GH-receptor antagonist does not feed back onto ghrelin secretion [116, 117]. Chronic GH excess or deficiency and exercise also do not consistently modify ghrelin production in humans or animals [118–120]. 

 Food intake, especially amino acids, glucose but not fructose, dodecanoate, and other long-chain fatty acids, T administration, euglycemic hyperinsulinemia, impaired glucose tolerance, infusion of free fatty acids, somatostatin, cortistatin or urocortin, obesity (elevated body-mass index and increased total, subcutaneous or visceral fat), weight gain, hyperthyroidism, and leptin injection suppress ghrelin concentrations [86, 97, 116, 117, 128–144]. A possible intracellular mediator of fasting and feeding's reciprocal control of ghrelin secretion is the mammalian target of rapamycin [145], which is suppressed by fasting [145]. In general, total ghrelin concentrations nearly double before meals and fall to a nadir about one hour thereafter [146]. Protein (more than lipid) ingestion strongly suppresses plasma acylghrelin, whereas carbohydrate initially suppresses and then elevates circulating ghrelin in humans [147]. Gastrectomy reduces total ghrelin concentrations by 65%–80% (to 20%–35% of baseline) [148], thereby verifying that the stomach is the major source of this hormone. 

 Acylated ghrelin typically changes in parallel with total ghrelin availability and in the fed state may rise recurrently before GH pulses [149]. Dissociations between acyl and desacyl-ghrelin levels occur after fiber versus total energy intake [150], intravenous glucose infusion [142], in renal failure [86], and in the portal vis-à-vis hepatic veins because of preferential liver extraction of the acylated moiety [142]. In clinical studies, meal-induced depression of ghrelin levels may be attenuated in children compared with adults, and in women with polycystic ovarian syndrome (PCOS) compared with healthy women, but accentuated in African-American compared with Caucasian women [151–154]. The antiandrogen, flutamide, increased ghrelin levels in patients with PCOS, suggesting suppression of ghrelin production via the androgen receptor in this hyperandrogenemic syndrome [152].

### 2.5. Metabolism of Ghrelin

**Figure 4:**
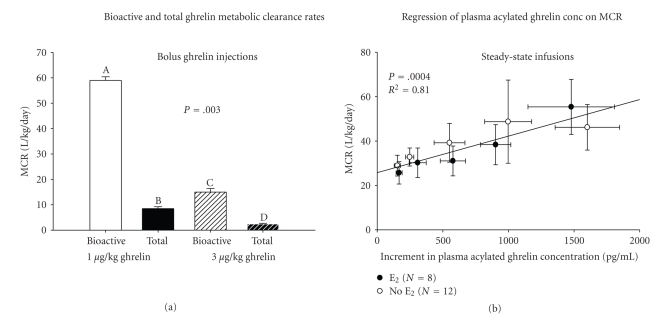
Strong impact of bolus ghrelin dose and ghrelin isotype (acylated [bioactive] or total ghrelin) on the metabolic clearance rate (MCR) of ghrelin in postmenopausal women. Means with different superscripts differ significantly by *post hoc *analysis after ANOVA (*P* = .003) (a). Linear relationship of steady-state MCR of acylated ghrelin to plasma acylghrelin concentration during constant ghrelin infusion (b). Adapted from [91] with permission.

The metabolic clearance rates of acyl and desacyl-ghrelin injected by bolus in humans differ by several-fold [91]: Figure 4(a). Active ghrelin (1 *μ*g/kg) is removed more rapidly (half-life 21 minutes) than unmodified ghrelin peptide (36 minutes) and partitions into a larger but finite distribution volume consistent with greater lipophilicity. At a higher ghrelin dose (3 *μ*g/kg), half-lives are 47 and 64 minutes for acylated and unacylated peptide. This concentration-dependence suggests interconversion of ghrelin isoforms and/or 2-compartment kinetics of acylated peptide [155]. Figure 4(b) illustrates that steady-state bioactive-ghrelin concentrations are not saturable at metabolic clearance rates of up to 60 L/kg/day. Acylated peptide binds to plasma high-density lipoproteins containing paraoxonase (an esterase) [156], but the size and fate of this reservoir are not known. An additional gastric ghrelin deacylation enzyme has been identified, lysophospholipase I [58]. The exact degree to which this enzyme regulates ghrelin biosynthesis secretion is still unknown. Desacyl-ghrelin appears to undergo significant renal clearance [86], whereas acylghrelin is extracted substantially by the liver [142, 157].

## 3. Ghrelin (GHS) Receptor-1a

The ghrelin receptor exhibits high (about 50%) basal constitutive activity [158, 159] and responds to inverse agonists, partial agonists, and allosteric antagonists [160, 161]. In particular, inverse agonists repress basal receptor activity, as defined by inositol-triphosphate, Ca^2+^, or diacylglycerol signal generation [159, 162]. Since blood ghrelin levels rise between meals and overnight, a ghrelin-receptor inverse agonist might be used to minimize hunger at these times and overnight [163]. In two families, short stature accompanied GHS-R1a mutations that reduced constitutive GHS-R1a activity [27], thereby implying biological relevance of basal receptor activity. 

 Multiple experimental strategies have been employed to test the biological impact of silencing ghrelin or GHS-R1a activity: Table 2. Consistent outcomes in animal models comprise loss of appetitive, locomotor, and somatotropic regulation by exogenous ghrelin; modest reduction of body weight, IGF-I concentrations, and GH pulses in the female animal; increased fat oxidation; a rise in mean arterial blood pressure; reduced obesity and improved glucose tolerance, but with a potentially higher risk of hypoglycemia during prolonged fasting; and decreased development of fatty diet-induced diabetes mellitus [164–168, 174–185]. Double transgenic knockout of ghrelin and cognate receptor is marked by diminished adult body weight, greater energy expenditure, and higher locomotor activity [169, 186]. Thus, GHS-R1a is a physiological mediator of ghrelin's stimulation of GH secretion, repression of oxygen consumption and locomotor activity, and enhancement of appetite. GHS receptor type 1b arises from a nonspliced transcript, whose product does not bind acylghrelin or confer known bioactivity [5, 187].

 Unlike ghrelin, synthetic GHS's acting via GHS-R1a typically do not require acylation and are not known GOAT substrates. Moreover, multiple biological effects have been reported for desacyl-ghrelin, which essentially does not bind GHS-R1a. A partial registry of effects comprises differentiation of skeletal muscle; relaxation of vascular smooth muscle; suppression of proinflammatory cytokines; inhibition of apoptosis of cardiomyocytes, pancreatic beta cells, and endothelial cells; antagonism of ghrelin's stimulation of somatic growth, hepatic gluconeogenesis, and appetite; hypotensive effects; repression of fatty-acid oxidation; and stimulation of adipogenesis [22, 75, 188–201]: Table 3. Although local acylation could explain certain actions of unacylated ghrelin, other effects that oppose or differ from those of acylated peptide raise the possibility of non-GHS-R1a mediation. Synthetic analogs of ghrelin likewise may exhibit partial agonism (e.g., stimulation of appetite but not GH release, and vice versa), antagonism, and inverse agonism despite similar GHS-R1a binding affinities [160, 202–207]. To date, no desacyl-ghrelin and no other GHS receptors have been cloned to explain such data [175]. 

 Acylghrelin, unlike the naked peptide, is a consistently effective agonist of GH secretion in multiple species from fish to mammals [34, 65, 215–221]. Nonacylated synthetic GHS can stimulate GH secretion in occasional models [222–224]. In fish, desacyl-ghrelin may function as an inhibitor of ghrelin's stimulation of appetite and locomotion [225]. In mice, transgenic overexpression of desacyl-ghrelin decreases food intake, gastric emptying, GH (female animal) and IGF-I (both sexes) concentrations, body weight and length, and GH release induced by exogenous ghrelin [75]. CNS delivery of desacyl-ghrelin in the rat likewise impedes food intake and gastric emptying [190, 196]. In humans, exogenous desacyl-ghrelin does not restrict ghrelin-induced GH secretion but disinhibits ghrelin's repression of insulin secretion [210, 221, 226]. Acylated ghrelin, unlike GHRH, does not induce GH-gene transcription. Exceptions include the pituitary glands of embryonic fish and prepubertal rats in vivo and ovine somatotropes in vitro [227–229]. Both GH and IGF-I can feed back to inhibit hypothalamopituitary stimulation by ghrelin/GHS, but feedback inhibition is less marked on GHS than GHRH stimulation [111]. Feedback appears to involve induction of periventricular hypothalamic somatostatin outflow [230], which quenches both GHRH and GH secretion [4].

## 4. Ghrelin and GHRH Synergy

Active ghrelin acts as an amplifier of GHRH, the primary agonist of GH secretion [4]. Human and murine hypothalami contain a wide network of ghrelin-expressing neurons, which extends across the arcuate, ventromedial, paraventricular and multiple other nuclei [8–11, 231–235]. Antisense RNA-mediated silencing of the murine GHS-1a receptor localized to GHRH neurons resulted in reduced adult weight, fat mass, pulsatile GH secretion, and IGF-I production in the adult female only [165]. Arcuate-nucleus GHRH-gene expression also declined in these animals [236], indicating that GHS-R1a not only transduces GHRH release but also maintains GHRH gene transcription [237, 238]. Consistent with this inference, GHS' stimulation of maximal (5-35-fold) GH release requires an intact hypothalamopituitary unit allowing GHRH outflow to the pituitary [239–242]. Accordingly, GHRH (−/−) mice, GHRH-receptor (−/−) mice, and rare patients with inactivating mutations of the GHRH receptor or congenital aplasia of the pituitary stalk are hyposomatotropic, and respond sparingly (<3-fold) to ghrelin/GHS [241, 243–246]. Immunoneutralization of GHRH and antagonists of GHRH also restrict GHS's effects markedly, resulting in responses of somatotrope cells to GHS similar in magnitude to those in inferred directly in vitro [1, 247]. 

 Ghrelin or synthetic GHS achieves synergy with GHRH (supraadditive stimulation of GH secretion) when a near-threshold dose of GHS is combined with a maximally effective dose of GHRH in the human, rat, pig, cow, and dog in vivo [248–255]. Synergism is not observed after combined stimulation with either maximal GHRH and maximal GHS or submaximal GHRH and maximal GHS stimulation [65, 254]. Synergistic stimulation of GH release is absent in pituitary cells cultured in vitro and in patients with destructive lesions that separate the hypothalamus and pituitary gland [65, 216, 219, 242, 250, 256, 257], thus defining a critical role for joint hypothalamopituitary effects. Nonetheless, precise mechanisms subserving synergy are not established. Proposed mechanisms include ghrelin-mediated (a) opposition to hypothalamopituitary actions of SS and/or (b) stimulation of an unknown (“U”) synergy factor [4, 29, 65, 151, 255, 258–260]. A substance like galanin or an endogenous opiatergic peptide might represent such a factor, since both peptides release and synergize with GHRH and their neurons innervate periventricular SS neurons [261–267]. Reduced pituitary action of SS is unlikely to be the sole potentiating mechanism subserving GHS action, since GHS and GHRH synergize even after immunoneutralization of SS [268]. Although GHRH can induce the pituitary ghrelin and GHS-R1a genes [42], the physiological impact of this potentially amplifying mechanism is not known.

 Since highly selective GHS-R1a antagonists are not available for clinical investigation, determining the exact extent to which endogenous ghrelin participates in amplifying GH secretion in human physiology, including *in utero*, in infancy, childhood, puberty, adulthood, and senescence remains difficult [4, 65, 250]. Nonetheless, mutational reduction of constitutive ghrelin-receptor activity in humans is associated with short stature [27]. Conversely, overexpression of neuronal GHS-R1a in female mice elevates GH and GHRH gene expression [28]. In addition, prolonged administration of ghrelin or synthetic GHS in humans augments GH, IGF-I and IGFBP-3 concentrations and lean-body mass, and diminishes total-body fat, while eliciting transient secretion of adenocorticotropin hormone (ACTH), cortisol, and prolactin [64, 269–273]. The last observation is significant, because higher doses of ghrelin/GHS evoke ACTH, cortisol, and prolactin secretion acutely in humans and animals, putatively by inducing hypothalamic secretion of one or both primary ACTH-releasing peptides, corticotrophin-releasing hormone (CRH), and arginine vasopressin (AVP) [272, 274–278]. How GHS induces prolactin release is not clear. Doubling or tripling plasma ghrelin concentrations evokes GH secretion in humans without measurably altering ACTH, prolactin, insulin, or free fatty-acid concentrations [279]. The basis for the dose-response difference and tachyphylaxis of the corticotropic, but not the GH-releasing, effect of GHS is not yet evident [280]. Likewise, why age and obesity impair GHS-induced secretion of GH but not of ACTH and prolactin is not known [113, 281]. 

 Depending upon chemical structure and dose, synthetic GHS can stimulate GH secretion after intranasal, oral, s.c., or i.v. administration [4, 65, 250, 272, 282, 283]. Entry of acylghrelin (but not desacyl-ghrelin) into and exit of the same from the brain is via structurally selective saturable transport [284–286]. Since hypothalamic GHRH release is prerequisite to maximal GH stimulation, GHSs have clinical therapeutic potential in treating: (i) idiopathic short stature in patients with preserved CNS outflow of GHRH [64, 287–290]; (ii) hyposomatotropism in aging, wherein GHRH release may be diminished but is not absent [271, 291, 292]; (iii) hyposomatotropism associated with visceral adiposity, including the HIV lipodystrophy syndrome [167, 179, 190, 293–295]; and (iv) conditions of partial GH resistance accompanying anorexia, cachexia, or heightened catabolism, such as metastatic cancer, hepatic or renal failure, chronic obstructive pulmonary disease, systemic inflammation, and advanced heart failure [133, 250, 296–298]. Preclinical data in these areas and clinical data in healthy subjects suggest the merit and feasibility of more comprehensive investigations [270, 271, 299–301], since prospective, double-blind, placebo-controlled trials are lacking. Critical issues to be resolved include long-term safety, duration, efficacy, and indications for GHS administration in selected GH-deficient states.

## 5. Multifaceted Actions

Short-term ghrelin/GHS administration stimulates GH secretion, locomotor activity, and appetite, increases plasma free fatty acids, imposes mild peripheral (muscle) insulin resistance, suppresses insulin secretion, inhibits fat oxidation, and promotes adipocyte glucose metabolism [4, 65, 170, 209, 210, 302–307]. Moreover, GHS is both antiproliferative and proliferative depending upon cell type [308–310]. These and other multifaceted actions of ghrelin are discussed next.

### 5.1. Appetitive Effects of Ghrelin

**Figure 5:**
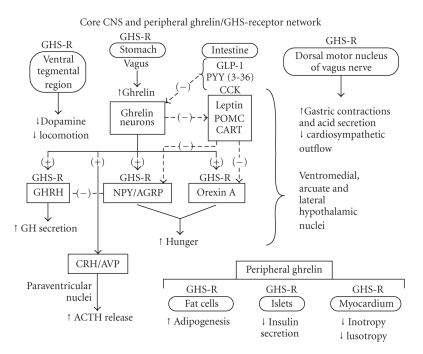
Basic ghrelin network influencing locomotion (*left upper quadrant*), gastrointestinal signals to appetitive and anorexigenic centers (*middle section*), gastric motility (*right upper quadrant*) or peripheral target tissues (*right lower segment*). Unpublished schema.

Ghrelin consistently enhances appetite by 25%–30% in fasting humans and animals with the possible exception of chicken, quail, and sheep [37, 39, 295, 311–315]. Endogenous ghrelin may enhance anticipatory motor activity, before scheduled meals [316]. Active ghrelin stimulates and suppresses glucose-sensitive neurons in the lateral and ventromedial hypothalamus, respectively, stimulating hunger and inhibiting satiety [317]. The orexigenic effect arises by combined activation of neuropeptide Y/agouti-related peptide (NPY/AGRP) and orexin A neurons [314, 318–322]. Concomitantly, acylghrelin antagonizes satiety-promoting and anorexigenic signals, such as leptin [38, 323], corticotropin-releasing hormone (CRH), cocaine and amphetamine-regulated transcript (CART), proopiomelanocortin (POMC), and alpha-melanocyte-stimulating hormone (alpha-MSH) [324–326]: Figure 5. Obestatin is not pictured because of its controversial role in appetite. AGRP is an endogenous inhibitor of alpha-MSH, thereby promoting positive energy balance. Ghrelin may act in part by inducing the intracellular signal mammalian target of rapamycin in AGRP neurons, a messenger which promotes protein synthesis and limits apoptosis [327]. Both NPY and AGRP participate in the orexigenic action of ghrelin, since transgenic disruption of both mediators is needed to quench appetite [303, 321]. The potent orexigen, orexin (hypocretin), which is a key peptide in maintaining wakefulness [328], is also involved in the appetitive action of ghrelin, in that orexin-A immunoneutralization or receptor silencing attenuates ghrelin's orexigenic action [329]. A key neuronal biochemical mediator of energy-depletion mediated appetitive drive may be the low-ATP sensing protein kinase, AMP kinase, which is stimulated by ghrelin and cannabinoids [330]. Desacyl-ghrelin exerts opposite effects, namely. decreases food intake, fat mass, and gastric emptying [190, 293]. Ghrelin also suppresses pancreatic beta-cell insulin secretion, which may contribute to orexigenesis, given that insulin can function as a CNS satiety signal [331]. 

**Figure 6:**
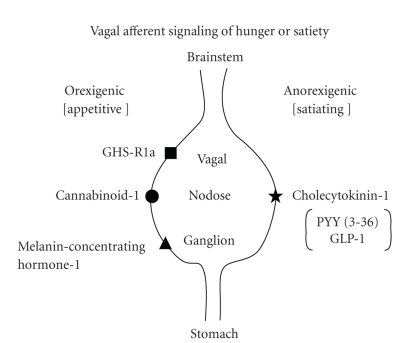
Complementarity of ghrelin's vagal-nerve signaling via GHS-R1a with that of other orexigenic (*left*) or anorexigenic (*right*) peptides. PYY: polypeptide YY; GLP-1: glucagon-like peptide. Unpublished sketch.

 The peripheral vagus nerve and its dorsal motor nucleus in the brainstem and ventral tegmental neurons mediate some of systemic ghrelin's appetitive effects [332–336]. Hunger-suppressing vagal afferent impulses from the stomach are subdued by ghrelin and by other orexigenic hormones, acting via the GHS-R1a, cannabinoid-1 (CB-1), and melanin-concentrating hormone-1 (MCH-1) and activated by anorexigenic receptors, like cholecystokinin-A (CCK-A), peptide YY (3–36), and glucagon-like peptide-1 [334, 337]: Figure 6. In fact, a CB-1 antagonist impedes ghrelin's appetitive effect, illustrating key facilitative interactions [338]. In addition, a CCK antagonist abolishes the capability of intraduodenal fat to suppress gastric ghrelin secretion [339], consistent with gastroduodenal feedback [340]. The dorsal motor nucleus of the vagus expresses GHS-R1a, which modulates limbic-system dopamine- and brainstem gaba, glutamine, and noradrenergic transmission to hypothalamic appetitive and satiety centers [335, 341–343]. Dorsal vagal neuronal GHS-R1a levels may decline with aging in the rat [344]. Ghrelin crosses the blood-brain barrier and directly inhibits leptin neurons and stimulates NPY/AGRP and orexin neurons [333], which are located, respectively, in the arcuate nucleu and lateral hypothalamus. MCH inhibits lateral hypothalamic neurons, providing additional feedback control [345]. Inhibition of brain (rather than exclusively peripheral vagal) GHS-R1a seems to explain the anorexigenic properties of ghrelin antagonists [346, 347].

 Although genetic disruption of GHS-R1a abrogates orexigenic stimulation by ghrelin, cachexia does not develop in the transgenic animals, including those with combined ghrelin and GHS-R1a knockout, putatively reflecting signal redundancy within nutritional networks [167, 353–355]. In particular, pathways mediating orexigenesis and satiety are convergent, oppositional, adaptive, and complex [186, 295, 319, 356–358], involving hormones from endocrine glands as well as gut mucosa and vagal afferent signals [35, 38, 359–362]. For example, intestinal mucosal L-cell-derived oxyntomodulin, a satiety signal, acts via the hypothalamus to inhibit vagally driven gastric secretion of acylghrelin, thus quelling hunger [363]. Plasma leptin and ghrelin concentrations tend to vary inversely in various clinical settings [364], and CNS leptin is a strong ghrelin antagonist [35], possibly by modulating NPY-Y1 or Y5 signaling [8, 38]. GHS-R1a is subject to systemic modulation, including upregulation by thyroxine and E_2_ and downregulation by glucocorticoids and GH [122]. In addition, E_2_ inhibits the acute orexigenic effect of exogenous ghrelin in the male and female rat [123]. Accordingly, the ghrelin system is a pivotal but nonexclusive member of a robust nutritional network in mammals [10, 294, 314, 319, 365–368]. However, ghrelin reduces food intake in neonatal chickens, possibly by elevating brain fatty-acid synthesis [369].

 GHS receptors are located in the stomach, gastrointestinal myenteric plexus, vagal nodose ganglion, dorsal motor nucleus of the vagus, arcuate-nucleus orexigenic neurons, such as NPY/AGRP and orexin A-expressing cells, anorexigenic neurons that are either leptin-sensing or leptin-producing, and multiple other unidentified neurons [314, 370, 371]. Silencing any one of NPY, AGRP or orexin-A-receptor genes limits but does not abolish ghrelin's orexigenic effect [329, 372–374]. Triple negation of NPY, AGRP, and orexin is necessary to eliminate ghrelin's appetitive effect [375]. Small interfering DNA-mediated neutralization of paraventricular neuronal GHS-R1a in rats imposes weight loss and reduces serum ghrelin without altering food intake, suggesting effects on energy expenditure as well [376]. Network robustness is conferred by the facts that ghrelin activates whereas leptin represses both NPY/AGRP, and orexin neurons; orexin A potentiates (feeds forward onto) NPY release via the orexin-1 receptor; NPY represses orexin release via NPY-Y1 receptors; neuropeptide W and galanin-like peptide further modulate orexin neurons [377, 378] NPY and orexin, respectively, suppress (feed back) and induce (feed forward onto) the potent anorexigens, leptin, and proopiomelanocortin, which in turn feed back on ghrelinergic cells [186, 235, 379–387]. Moreover, NPY and ghrelin neurons synapse on themselves, suggesting autofeedback-dependent regulation [235]. Collective feedforward/feedback circuits presumably subserve enhancement and suppression of food intake in a manner defined by sex, species, age, physical activity, ultradian rhythmicity, and sleep-wake cycles [35, 231, 295, 328, 388]. For example, sleep curtailment may augment appetite by simultaneously lowering circulating leptin and elevating ghrelin levels. The latter correlates with meal initiation [389]. These and other experiments illustrate the complex adaptability of nutritionally regulated neural networks [234, 278, 353, 362, 390]. 

**Figure 7:**
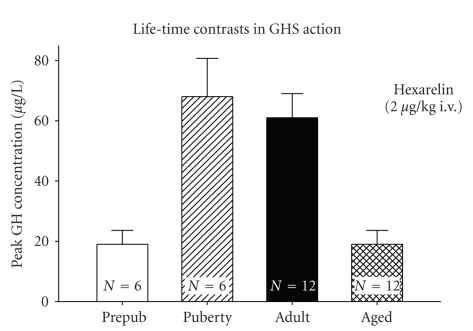
Lifetime variations in GHS (hexarelin) action to induce GH secretion in prepubertal, pubertal, adult, and aged humans (redrawn with permission from [488]).

 A 23-amino-acid C-peptidyl fragment of preproghrelin, named *obestatin*, has not fulfilled its original nomenclature as an antagonist of ghrelin's orexigenic effects [394–399]: Figure 7. In addition, transgenic knockout of the proposed obestatin receptor, GPR39, does not affect food intake [394, 396, 400, 401]. Whereas obestatin (probably better termed, ghrelin-associated peptide) does not alter pituitary hormone secretion [402], this peptide may suppress thirst, promote beta-cell survival and regeneration, activate urocortin-2 pathways and thereby inhibit gastroduodenal peristalsis, and induce early-response genes in gastric mucosa and preadipocytes [403–410]. A physiological distinction pertinent to CNS actions of ghrelin, but not obestatin, is that only ghrelin exhibits specific saturable binding to and endocytosis by cerebral microvessel endothelial cells, thereby allowing measurable permeation of the blood-brain barrier [284, 286]. However, obestatin attenuates the hypothermic effect of preproghrelin gene deletion [411].

 Transgenic overexpression of CNS and gastric ghrelin causes hyperphagia, increases energy expenditure, and decreases sensitivity to insulin and leptin in mice [170]. The orexigenic action of ghrelin does not seem to wane in this model, since adult transgenic animals continue to eat excessively after fat depots reach a maximum [412]. In a second model, 8 weeks of GHS treatment maintained orexigenesis in the rat [413]. In a third model, overexpression of GHS-R1a in GHRH neurons increased organ and muscle weight, while decreasing body fat in female animals [28]. Conversely, in a fourth model, transgenic reduction of GHS-R1a expression on GHRH neurons suppressed both GHRH and NPY gene expression, thereby diminishing GH and fat mass in the female [236]. In patients with renal or cardiac failure or chronic lung disease and cachexia, short-term (up to 3 weeks) administration of ghrelin once or twice daily may stimulate appetite and weight gain as assessed in uncontrolled studies [298–300, 414, 415]. However, in the perioperative orthopedic setting, ghrelin infusions for 3 weeks did not improve overall functional physical performance [416]. Nonetheless, the precise extent to which GHS agonists and antagonists will alter hunger or satiety over the longterm remains difficult to forecast [74, 232]. This issue is important since studies with leptin, an anorexigenic adipokine that antagonizes ghrelin, revealed tachyphylaxis to satiety-promoting effects [186, 417]. 

 Ghrelin itself undergoes tissue-specific regulation by short-term fasting, hypoglycemia, and nutrient deprivation. These factors depress brain ghrelin expression, while promoting gastric ghrelin secretion and amplifying CNS responses to ghrelin [9, 418, 419]. In species in which desacyl-ghrelin can antagonize the orexigenic effects of acylghrelin [293], regulation of GOAT activity in both the stomach and brain may be important to appetitive homeostasis [191, 196]. GHS analogs that do not require acylation have the merit of bypassing GOAT [420]. 

 Gastric-bypass procedures, especially when combined with truncal vagotomy, often reduce serum total ghrelin concentrations initially [426, 427]. In principle, reduced ghrelin availability would further attenuate adipogenesis [191, 195, 199]. However, in some studies prolonged weight loss after gastric bypass had no effect on fasting ghrelin levels [250, 428, 429] or reduced only acylated ghrelin [430]. In rodents, vagotomy impedes both appetitive and GH-stimulating effects of ghrelin [334, 336, 431, 432]. In humans, pharmacologic or surgical interruption of vagal cholinergic traffic lowers ghrelin concentrations and blunts appetitive stimulation but does not block GHS-induced GH release [336, 431, 433].

### 5.2. Insulinostasis

Ghrelin inhibits insulin (beta cells) and somatostatin (delta cells) but stimulates glucagon (alpha cells), secretion in vivo and in isolated pancreatic islets in vitro [304, 434–439]. Some early studies reported that GHS induces insulin secretion [440]. Inconsistencies may reflect the capabilities of desacyl-ghrelin to suppress hepatic glucose production and/or block acylghrelin's inhibition of insulin secretion [198, 221, 434, 441]. Other studies did not control for the fact that acutely elevated GH and glucose concentrations (induced by GHS) stimulate insulin secretion acutely. GHS receptors are expressed by both beta and alpha cells [442]. Ghrelin's inhibition requires Gi, which activates outward K^+^ currents and disables inward Ca^2+^ flux [443, 444]. These effects particularly impede rapid first-phase insulin release [444]. Moreover, acylghrelin antagonizes peripheral insulin action (muscle > liver), which effect is reversed by desacyl-ghrelin in GH-deficient adults [210]. However, desacyl-ghrelin has little if any effect on glucose, insulin, free fatty acid, or GH releases in (obese) humans [445].

 From a reciprocal perspective, insulin and somatostatin repress, whereas glucagon induces the gastric ghrelin gene in the rat [88, 124, 130]. In humans, glucagon injection inhibits ghrelin secretion [121], but via uncertain pathways. In rodents, insulin represses not only gastric but also CNS preproghrelin expression [446]. In accordance with insulin's negative effects, hyperinsulinemia associated with hepatic or skeletal-muscle insulin resistance and/or obesity correlates inversely with fasting ghrelin concentrations [71, 447, 448]. An exception is the genetic Prader-Willi syndrome marked by obesity, mild insulin resistance, and unexplained hyperghrelinemia with increased numbers of gastric ghrelin-expressing cells [449–451]. 

 Adult pancreatic islets express relatively little ghrelin protein and few GHS receptors, but fetal and neonatal islets exhibit abundant ghrelin gene transcripts and peptide in epsilon cells [452–454]. Although both acyl and desacyl-ghrelin promote beta-cell regeneration [408, 455], endogenous pancreatic ghrelin inhibits beta-cell function, since perfusion of the pancreas with ghrelin antiserum augments insulin secretion [408, 438, 444, 456]. In addition, genetic knockout of ghrelin and administration of GHS-receptor antagonists diminish fasting hyperinsulinemia and enhance glucose tolerance in rodent models [205, 439, 443, 444]. Thus, in knockout ghrelin mice, increased constitutive ghrelin-receptor activity may result in increased insulin secretion, which would be susceptible to inhibition by a GHS receptor antagonist with inverse agonist activity. Further investigations are required to appraise the influence of diet, age, gender, species, and obesity on pancreatic actions of ghrelin, given the high importance of drug development in this area. A desirable ghrelin antagonist would limit appetite, disinhibit insulin secretion, and minimize adiposity (by favoring fat oxidation) in patients at risk for the metabolic syndrome or type II diabetes mellitus [84, 205, 223, 457, 458]. Table 4 highlights some of these issues and identifies potential adverse consequences of prolonged GHS-receptor blockade.

### 5.3. Cardiovascular Effects

At doses that induce maximal GH secretion, ghrelin and GHS can exert direct vasodilatory, cardiotropic, and brainstem-mediated hypotensive effects, typified by a 10%–20% decrease in mean arterial blood pressure and an increase in left-ventricular ejection fraction [14, 183, 201, 459–464]. Desacyl-ghrelin also causes vasodilation and opposes cardiomyocyte and endothelial apoptosis [194, 465]. Conversely, a ghrelin antagonist elevates heart rate and arterial pressure in the conscious rat [183]. The mechanisms of ghrelin's effects include central suppression of sympathetic cardiac drive, systemic vasodilation, antagonism of angiotensin II and endothelin-induced vasoconstriction including of the pulmonary arteries, and potentiation of nitric oxide-mediated relaxation of vascular smooth muscle [14, 20, 197, 465–471]: Table 4. GHS receptors exist on endothelium, vascular smooth-muscle cells, and cardiomyocytes [13, 201, 472, 473]. Furthermore, ghrelin transcripts and protein are expressed in endothelial cells and cardiac muscle [466, 472]. Both ghrelin and desacyl-ghrelin reduce the rate of myocardial tension generation (negative inotropic effect) and the rate of tension relaxation (negative lusitropic effect), possibly in part independently of the GHS-1a receptor [13, 201, 330, 474]. Obestatin does not initiate these responses [475]. The membrane glycoprotein, CD36 initially recognized as a macrophage type B LDL-scavenger receptor and recently as a fatty-acid binding protein, may transduce or modulate certain cardiovascular and anti-inflammatory effects of ghrelin [188, 207, 474]. 

 The capability of ghrelin to limit apoptosis of endothelial cells and cardiomyocytes in vitro introduces the possibility of reducing endothelial dysfunction associated with atherosclerotic risk [476–478] and salvaging myocardium in zones of marginal chemotoxicity [479] or ischemia [22, 194, 461, 480]. Desacyl-ghrelin shares cardioprotective effects, but the mechanism is not known [194]. Increased coronary-artery perfusion pressure, reduced cardiac sympathetic activity, antagonism of L-type Ca^2+^ channel-mediated contractility, and activation of low-energy AMP-kinase may contribute to cardiotropism [461, 481, 482]. Resultant effects are reduced left-ventricular endsystolic volume and increased cardiac output [414, 460]: Table 5. How much of this benefit is due to stimulated secretion of GH, which exerts cardiotropic effects, is not known [483].

 Elevated ghrelin concentrations in patients with chronic heart failure may indicate a compensatory mechanism in particular [484] or reflect partial tissue resistance to ghrelin in cachetic states more generally [83, 485]. Three- and four-week pilot studies of exogenous ghrelin's anabolic and anticatabolic effects in chronic cardiac cachexia in the rat and human seem favorable [194, 301, 461, 486], although parallel-cohort prospectively randomized double-blind controlled clinical studies are not available [414]. Collective data invite more rigorous interventional studies in experimental models of both early and advanced myocardial injury and failure. In addition, further investigations are necessary to assess how GHS affects perfusion of the brain, skeletal muscle, kidney, liver, and intestine [296, 487].

### 5.4. Gastric Motility

Ghrelin is expressed in granules in gastric X/A-like oxyntic cells [489, 490]. The same granules contain immunoreactive motilin, a comparably strong but distinct prokinetic peptide [491, 492]. Obestatin is additionally present in many of the same cells, but its gastrointestinal function is not known [189, 404]. In one report, obestatin was able to elicit pancreatic zymogen secretion [493]. In other contexts, desacyl-ghrelin and obestatin, unlike ghrelin, inhibit gastric antral contractions [189]. Ingestion of medium-chain triglycerides in the infant or adult enhances gastric acylghrelin concentrations [494, 495]. Inactivation of prohormone convertase 1/3 (a preproghrelin endopeptidase) also increases ghrelin gene transcripts, suggesting negative feedback by ghrelin on its own transcription [496]. Fasting induces the preproghrelin gene in fish and mammals via efferent vagal cholinergic signals [497–499]. The cephalic (meal-visualization) phase of digestion in humans clearly depends on vagal efferents, which transduce prandial inhibition of ghrelin secretion [500]. In sheep, cholinergic blockade stimulates stomach ghrelin output [311], suggesting baseline cephalic phase-like inhibition in this species. 

 Motilin and ghrelin receptors are 36% homologous in peptide sequences; yet each is activated principally by the homologous agonist at physiological concentrations [492, 501, 502]. Further unlike GHS-R1a, the motilin receptor exhibits little constitutive activity. The GHS-R1a gene is conserved with 58% nucleotide homology in puffer fish and humans [63]. The receptor is present in the myenteric (neuronal) plexus, gastroduodenal mucosa, smooth muscle, and vagal nodose ganglia [503, 504]. Ghrelin stimulates smooth-muscle contractions and type III primary migrating-motor complexes in the stomach and proximal duodenum of the eel, rat, rabbit, mouse, guinea pig, and human; inhibits postprandial contractions (dog) and gastric accommodation (decreases residual stomach volume); and promotes gastric-acid secretion, which facilitates protein hydrolysis and Ca^2+^ absorption [505–511]. Current promotility drugs for the treatment of idiopathic, postvagotomy and diabetic gastric atony, and morphine-induced postoperative ileus include serotoninergic-3 agonists, dopamine antagonists, and motilin-receptor agonists, like erythromycin [492, 512–514]. Whereas desacyl-ghrelin may block certain promotility effects of ghrelin [208], ghrelin is unique in acting on all four of gastroduodenal myenteric neurons, smooth-muscle cells, the vagus nerve, and brain GHS-1a receptors to augment gastric emptying and lower pH [333, 512]. An additional effect is gastroprotection against alcohol and ischemic mucosal stress [515]. Histamine-2 agonists and gastrin synergize in promoting preprandial acid secretion [516, 517], thus illustrating intragastric interactions with ghrelin. 

 Preclinical and clinical data indicate that GHS-receptor agonists can accelerate gastric emptying even in the presence of autonomic denervation, and reduce postoperative gastrointestinal ileus [492, 506, 509, 511, 512, 518–522]. Patients with diabetic gastroparesis may have low plasma total ghrelin levels, possibly due to vagal denervation and/or chronic glucagon excess [121, 523, 524]. What is unclear is the risk/benefit ratio of systemic GHS treatment in patients with GI bleeding, autonomic neuropathy, and/or concurrent postoperative needs for narcotics, anticholinergics, and other drugs. Concerns would include possible hypotension due to GHS-mediated relaxation of vascular smooth muscle and negative cardiac inotropy and possible deterioration of glucose tolerance due to suppression of insulin secretion and augmentation of hepatic glucose output [198]. Thus, high agonist selectivity will be necessary to ensure clinical safety. 

### 5.5. Sleep-Wake Regulation

Ghrelin concentrations rise during the first four hours of normal sleep in humans [525]. The mechanism is not known. Viewed conversely, ghrelin administration enhances slow-wave (non-REM) sleep including in young and older men but not women [526–530]. In one clinical study, the GHS peptide, hexarelin, reduced non-REM sleep whereas GHRP-6 increased the same, suggesting GHS-receptor pleiotropy or multiplicity [531]. How ghrelin can enhance deep sleep and yet activate orexin-A neurons [328], which are coupled to arousal-wakefulness and appetite, is not yet clear [328, 532]. In addition, why patients with obstructive sleep apnea, albeit obese, have elevated acylghrelin concentrations has not been elucidated [533]. However, reduction of hypoxic episodes decreased ghrelin, suggesting that ghrelin is a stress-responsive hormone.

### 5.6. Cell Survival and Cachexia

Ghrelin and GHS analogs exert differentiative as well as proliferative and antiproliferative effects in vitro and in vivo [193, 211, 455, 534]. Examples include mitogenic and antiapoptic actions on pancreatic islets, spinal-cord, cortical and brainstem neurons, osteoblasts, fetal lung branches, endothelium, cardiomyocytes, and adipocytes on the one hand, and apoptotic effects in certain adrenal, lung, and prostate cancer cell lines on the other hand [15, 21, 22, 192, 211–213, 309, 391, 480, 535–541]. Several cell-cycle effects can be induced by incubation with either acyl- or desacyl-ghrelin, raising the possibility of involvement of both GHS-R1a and non-GHS-R1a receptor pathways [58, 537]. This reflects the fact that GHS-1a is essentially unresponsive to desacyl-ghrelin [22, 177]. Short-term ghrelin/GHS administration to enhance neuronal regeneration after ischemic or toxic insults thus represents another major point of potential therapeutic focus [214, 542, 543]. Because ghrelin can also stimulate proliferation of certain carcinoma cell lines in vitro [308, 309, 544], studies to exclude longterm oncogenic effects are needed. 

 Inasmuch as appetite wanes in the cachectic stage of carcinomatosis, ghrelin is being evaluated as an anticachectic agent. Studies in the rat, mouse, and human indicate that ghrelin/GHS administration can dose-dependently enhance appetite, body weight, and fat mass in several short-term models of disseminated neoplasia [545–549]. Caveats are that rigorous controls and blinding are often lacking, and evaluations are necessarily of short duration initially. In addition, some studies do not show orexigenic benefits [550, 551]. A preliminary study in 12 cachectic patients with dialysis-dependent kidney failure showed appetitive enhancement by ghrelin compared with randomized double-blind placebo injection over a 1-week interval [415].

### 5.7. Adipogenesis

Prominent effects of ghrelin/GHS include direct promotion of preadipocyte proliferation and adipocytic differentiation and hypertrophy [76, 552]: Table 6. GH-independent CNS actions may participate in lipogenesis [553]. Desacyl-ghrelin shares several of these actions [191, 199]. Relevant mechanisms embrace repression of insulin-sensitizing genes, such as adiponectin, and induction of adipocyte leptin and peroxisome-proliferating activator receptor- (PPAR-) gamma genes; stimulation of endothelial lipoprotein lipase, adipocyte fatty acid synthase, acetyl CoA carboxylase, and fat-cell glucose uptake; and inhibition of fat oxidation via rate-limiting carnitine-palmitoyl transferase [167, 171, 172, 191, 554, 555]. As a more general metabolic mechanism, ghrelin mediates inhibition of sympathetic outflow to thermogenic fat depots, reduction of uncoupling protein-1 expression, and thereby decreased resting energy expenditure [556, 557]. Species appears to influence some mechanisms. For example, in sheep, ghrelin potentiates glucose-induced insulin secretion, which is antilipoytic [558]. In rodents, unacylated ghrelin can elevate insulin concentrations, which is adipogenic [441]. Neither is the case in humans [210]. 

 CNS pathways may participate in stimulating adipocyte hypertrophy [171] and increasing the respiratory coefficient (increased ratio of glucose/fatty-acid oxidation) [234, 392, 555]. In particular, ghrelin acts in part by repressing leptin, CRH, and histamine (anorexigenic) and activating orexin and NPY (appetitive) signaling [184, 559–561]. Orexin A in turn inhibits GHRH gene expression, thus limiting GH-dependent lipolysis [562]. Thus, orexin contributes to fat accumulation by augmenting appetite, attenuating satiation, and restricting GH secretion [38, 171, 443]. 

 Despite ghrelin's strong adipogenic effects, subcutaneous, visceral, and total adiposity correlate negatively with blood ghrelin concentrations [116, 563]. This may be because a significant longterm effect of ghrelin is to drive pulsatile GH secretion, which is strongly lipolytic [4]. Animal models indicate that ghrelin antagonists are able to reduce hyperglycemia and adipogenesis, enhance energy expenditure and fat oxidation, stimulate insulin secretion and action, and heighten resistance to diet-induced obesity [167, 168, 179, 182, 184, 205, 564]. A potential risk of prolonged antagonist administration in the fasting state could be endogenous insulin-induced hypoglycemia [166], although this adverse event has not been observed in humans.

### 5.8. Bone Formation

GHS-R1a and ghrelin peptide are synthesized in bone cells, such as chondrocytes and osteoblasts [12, 565]. Albeit moderately well understood in other tissues, mechanisms mediating regulation of GHS-R1a in bone remain unclear: Table 7. Acylated and unacylated ghrelin stimulate osteoblast proliferation and differentiation, increase osteoblastic markers like osteocalcin and bone alkaline phosphatase, and repress osteoblast apoptosis putatively via phosphatidylinositol 3-kinase and mitogen-activated protein kinase signaling [566]. Moreover, in GH-deficient rodents, ghrelin administration augments Ca^2+^ retention in the skeleton, thereby elevating bone-mineral density [565]. The clinical impact of these effects is not yet established.

### 5.9. Stress Adaptations

Recent studies point to a role for ghrelin in modifying pathophysiological adaptations to stress [275, 567, 568]. For example, ghrelin concentrations rise acutely after major surgery along with inflammatory cytokines like tumor-necrosis factor-alpha and interleukin (IL)-6 [569]. In mice, transgenic knockdown of the ghrelin receptor accentuates adverse effects of chronic social-defeat stress [570]. Analogously, ghrelin may permit adaptation to pain, since it blocks spinal-cord nociceptive signals [571]. Lipopolysaccharide endotoxin stress lowers blood ghrelin levels, causing an associated reduction in gastric emptying [572]. The latter is substantially relieved by ghrelin infusion. Under some conditions, desacyl-ghrelin inhibits gastric contractions by activating stress-adaptive CRH receptor-2, for which urocortin is the natural ligand [208, 348]. Ghrelin also induces the anorexigenic hypothalamic CRH gene [567], which normally stimulates ACTH release. Whether CNS-mediated inhibition of food intake by desacyl-ghrelin proceeds by activating the CRH pathway is not known [196]. 

 Acute vascular-endothelial biochemical stress responses are modified by ghrelin. This peptide induces nitric oxide synthase (NOS) and represses the generation of reactive oxygen species, thereby attenuating endothelial injury [20, 573]. The protective effects require GHS-R1a, phosphotidylinositol-3 kinase, and Akt/protein kinase B [20]. A long-term action of ghrelin may be to retard endothelial apoptosis [574]. Ghrelin can also directly stimulate human vascular endothelial-cell migration, but the impact of this effect is not so clear [575]. Substantial additional work is needed to extend understanding of these aspects of ghrelin pathophysiology.

### 5.10. Immune Modulation

Ghrelin, GHS receptors, GH, and GH receptors are expressed in human monocytes and B and T lymphocytes [576]. In general, ghrelin's effects are antiinflammatory and immune-enhancing, for example, diminution of monocytic, bacterial and endothelial inflammatory factors driven by endotoxin exposure, sepsis, arthritis, interleukin 1 and 6, tumor necrosis factor-alpha, and nuclear-factor kappa B [200, 577–579], and stimulation of proliferation of thymic epithelial cells and T lymphocytes [580, 581]. Ghrelin also suppresses neutrophil and macrophage migration, caspase activation, reactive oxygen-species generation, and endoplasmic-reticular stress activation [582–584]. The combined impact may be to restrict lethal hepatic and pulmonary microvascular injury in sepsis [515, 585–587]. Ghrelin concurrently induces antiinflammatory cytokines, like interleukin-10 [578], and augments organ perfusion pressure in sepsis [515, 588]. Sepsis-associated gastric mucosal injury, an additional cause of mortality due to hemorrhage, is prevented in some models [589]. Certain of these effects may require the vagus nerve [590]. In acute renal failure, ghrelin treatment can repress inflammatory cytokines in the blood and brain, limit protein catabolism, enhance food intake, and attenuate renal injury due to endotoxemia and ischemia [415, 591–594]. Total but not acylghrelin concentrations are elevated in endstage renal failure [595] and normalize following kidney transplantation.

 Long-term surveillance of ghrelin and GHS receptor-deficient and ghrelin-overexpressing transgenic animals will be needed to assess whether prolonged changes in ghrelin availability influence immune function or inflammatory disease.

### 5.11. GHS Administration in Protracted Critical Illness

Although acute stress elevates GH secretion [596], extended critical illness suppresses all three of GH, IGF-I, and IGFBP-3 concentrations and inhibits tissue actions of GH. In unfed patients with multiorgan failure, total ghrelin levels rise [86, 131]. Infusions of GHS alone or combined with GHRH substantially reverse biochemical markers of hyposomatotropism in this setting [597, 598]. However, recovery from multiorgan failure and inflammation is necessary to alleviate tissue resistance to GH [4]. In animal models, ghrelin administration reduces sympathetic outflow, acute renal failure, inflammation in the lung, stomach and liver, and lethality of sepsis [200, 344, 515, 586–590, 599–601]. Contrastingly, glucocorticoid deficiency, and glucocorticoid excess alter the GHS axis by, respectively, diminishing blood ghrelin and brain GHS-R1a levels [421, 602, 603]. Further investigations are needed in this meritorious area.

### 5.12. Ghrelin in Pregnancy and Lactation

Ghrelin is expressed abundantly by the placenta [604], and ghrelin can stimulate GH release by human fetal pituitary cells in vitro [605]. Immunoneutralization of maternal ghrelin in the rat diminishes fetal body weight, raising the possibility of maternal-fetal exchange of ghrelin [606]. Maternal ghrelin and placentally derived variant-GH concentrations in the human peak at about 18 and 34 weeks gestation, respectively, fall thereafter, and reach a nadir approximately 3 days postpartum [607–609]. The decline in ghrelin in the third trimester of pregnancy is inversely related to blood pressure, resistin, and TNF-alpha levels [95, 610]. Although human umbilical-cord acylghrelin levels exceed those in the mother, the role of placental ghrelin is not well understood [611]. Placental insufficiency, low birth weight, and maternal fasting elevate fetal ghrelin levels [611, 612]. Postpartum acylghrelin concentrations increase by several-fold over midpregnancy values [613], but GH responses to GHS are reduced in breastfeeding women, especially in the hyperprolactinemic setting [614]. The basis may involve pregnancy-associated suppression of hypothalamic GHRH and GHS-R1a with reciprocal induction of the SS gene due to feedback by high placental somatomammotropin (GH isotype V) and maternal IGF-I concentrations [422]. 

 In the rat, lactation induces both hypothalamic and pituitary GHS receptors [615]. Pituitary GHS-R1a is maximal in the newborn pup and pubertal animal [423]: Table 7. Estradiol, T4, and GHRH may contribute to these maxima, since the GHS-R1a promoter is responsive to estrogen and cyclic AMP [122, 187]. Ghrelin is secreted into colostrum and milk, but its effect on the suckling infant is not well delineated [616]. In one preclinical study, treating rat pups with ghrelin reduced pancreatic exocrine development before weaning and exerted the opposite effect after weaning [617].

### 5.13. Antireproductive Effects of GHS

Exogenous ghrelin reduces LH pulse frequency in the adult male rat, cyclic or gonadectomized female rat, and ovariectomized monkey [618–622]. Ghrelin may also inhibit embryo development, decrease litter size, and delay pubertal onset in the male rat [623, 624]. The mechanism may involve neuropeptide Y (Y1 or Y5) or CRH-dependent inhibition of kisspeptin, which together supervise gonadotropin-releasing hormone secretion, and thereby pulsatile LH secretion [622, 624–627]. Other sites of inhibition may include uterine epithelium (by inducing IGFBP-1 and lowering free IGF-I), Leydig cells in the testis, and granulosa-luteal cells in the ovary (by blocking steroidogenesis) [624, 628–633]. Although physiological effects are not clear, ghrelin is expressed in sheep oocytes [634], and GHS-R1a in Sertoli (nurse) cells in spermatogenic tubules [623]. Further study is required to verify these inferences and evaluate clinical relevance [623]. For example, ghrelin infusion had no demonstrable effects on LH secretion in the early follicular phase of the menstrual cycle [635].

### 5.14. Neuroendocrine Tumors

Both ghrelin and obestatin are detected in various neoplasms, such as enterochromaffin tumors (e.g., carcinoids), pituitary adenomas, and carcinoma of the pancreas, lung, and breast [406, 636, 637]. The amount of peptide secreted is usually insufficient to serve as a tumor marker or to elicit clinical symptoms or signs.

### 5.15. Body-Composition Effects

**Figure 8:**
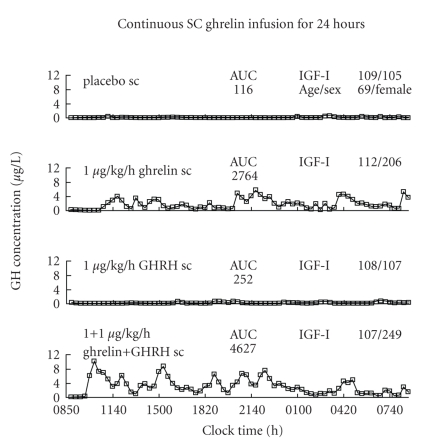
Continuous subcutaneous (SC) infusion of saline, GHRH or ghrelin, or both (1 *μ*g/kg/hour) for 24 hours in a normal 69-year-old woman. Data are 20-minutes GH concentrations (*y*-axis) plotted against time (*x*-axis). AUC: area under the GH versus time curve. IGF-I concentrations at the start and end of each infusion are stated in the upper-right corner of each panel in units of *μ*g/L.

Clinical studies in older individuals indicate that prolonged (up to 1 year) administration of GHS orally can increase lean-body mass, decrease abdominal visceral fat, and possibly improve certain performance measures, such as stair climbing and timed walking [548, 638]. In analyses comprising 1-to-30 days of parenteral GHS delivery, IGF-I concentrations also rise, but cortisol and prolactin do not [270–272]: Figure 8. Adverse events included insomnia, fatigue, small increases in fasting glucose or glycated hemoglobin, and mild insulin resistance [638]. An important consideration is that acute diabetogenic effects of GHS may be attenuated by its longterm antidiabetic effects: Table 8. Insomnia could reflect GHS's stimulation of orexin pathways, whereas glucose intolerance could reflect insulinostasis and insulin resistance due to acute free fatty-acid release in humans [306]. Short-term use of ghrelin in severe cachexia enhanced appetite and/or weight gain in some studies [298, 300]. Whereas both glucocorticoid deficiency and excess in chronic diseases can reduce GH response to GHS [4, 421], combined GHRH and ghrelin infusion is more effective at driving GH secretion than ghrelin alone [270]: Figure 8. Viral vector delivery of ghrelin augmented weight in the rat [639], introducing an additional potential avenue of treatment beyond subcutaneous, intravenous, oral, or intranasal administration [282].

 A small percentage of GH-deficient adults (10%) also respond acutely to GHS, suggesting some preservation of somatotrope function and GHRH availability [640]. In other settings, injected GHS showed high specificity (95%) but low sensitivity (80%) in detecting GH deficiency [641]. Combining GHS with GHRH and/or L-arginine improves test sensitivity [4, 163].

### 5.16. Species Differences

Species differences in ghrelin structure, and to a lesser degree ghrelin action, have been articulated [2, 26, 62, 276, 315, 642]. In the eel, ghrelin-21 predominates instead of ghrelin-28 [643]. In fish, decanoyl rather than octanoyl ghrelin stimulates food consumption and increases liver and fat mass [644].

### 5.17. Genetic Considerations

Epidemiological studies have not identified common genetic haplotypes of GHS-R1a, which predispose to obesity or short stature [645]. Two-weak associations of GHS-R1a polymorphisms with metabolic syndrome or cardiovascular risk require confirmation [646, 647]. Similar preliminary data apply to polymorphisms of the ghrelin gene [393, 648].

## 6. Summary

In addition to increasing GH, as the term *ghrelin *implies, GHS regulates inflammation, cellular proliferation, apoptosis, differentiation, and hormone secretion via receptors located in the brain, stomach, intestine, heart, arterial wall, bone, fat cells, and pancreas (exocrine and endocrine). Acylated and unacylated ghrelin can exert both similar (antiapoptotic) and opposite (appetitive) effects. Promising clinical applications of ghrelin agonists and antagonists arise in relation to metabolic, gastric, GH-stimulating, anti-inflammatory, and cardiotropic effects. Nonetheless, there are both desirable and potentially undesirable aspects of chronic administration of ghrelin agonists or antagonists: Table 9. Accordingly, substantial further advances in ghrelin biology will be important.

## 7. Speculations

A saga is evolving within the ghrelin system due to the recent identification of the ghrelin O-acyl transferase (GOAT) enzyme [47, 48]. The octanoyl addition is of major biochemical and functional significance. Not only is ghrelin the first natural hormone with a fatty-acid addition, but also octanoylation is essential for binding and activating the receptor, GHS-R1a, probably by determining the active receptor-specific conformation of the ghrelin 1–28 molecule. Final isolation of the GOAT enzyme may not be so easy, since it is tightly bound by 12 transmembrane domains spanning the endoplasmic reticulum (ER) [47].

 Important issues arise regarding the chemistry and biology of posttranslational octanoylation of ghrelin hormone. The current concept is that preproghrelin 1–117 or proghrelin 1–94 is octanoylated for fundamental biological reasons. In consonance with this postulate, production of octanoylated ghrelin 1–28 is restricted to the conjoint anatomical and intracellular sites of GOAT and preproghrelin 1–117 biosynthesis. Furthermore, if the desacylated ghrelin 1–28 is not directly octanoylated by GOAT, the specificity of biological regulation is made even more precise. Available evidence indirectly suggests that GOAT first octanoylates the preproghrelin 1–117 or the proghrelin 1–94 molecule. In the former case, octanoylation would occur cotranslationally rather than posttranslationally. This would require an integrated cleavage of the signal peptide. Subsequently, several prohormone convertases (PC1/3, PC2, and furin) permit sequential formation of octanoylated Ser^3^ proghrelin 1–94 and octanoylated Ser^3^ ghrelin 1–28 [496, 649, 650]. 

 Before the identification of GOAT, Zhu, Cao, Voogd, and Steiner published the finding that 1–94 proghrelin was octanoylated, whereas ghrelin 1–28 remained unoctanoylated [496]. These results support the inference that octanoylated Ser^3^ ghrelin 1–28 is derived from octanoylated proghrelin 1–94. The specific actions of convertases are now being resolved. Zhu et al. demonstrated that PC1 must be the enzyme that cleaves proghrelin in vivo, because only proghrelin and not processed ghrelin is made in PC1-knockout mice [496]. In addition, furin can cleave proghrelin in vitro as described in the laboratory of Lindberg and, more recently, that of Kojima in transfection studies [72, 649]. Why furin does not suffice in the PC1 gene-deletion model is not clear. 

 In addition to GOAT, another human ER oxyesterase has been purified and characterized from a stable human erythroleukemia cell line by Ozawa, Speaker, and Lindberg [56]. The acronym of this additional oxyesterase is ERAT for endoplasmic reticulum O*-*acyl transferase. ERAT will esterify modified proghrelin having an N-terminal tripeptide extension, but not the proghrelin 1–94 molecule. Notably ERAT octanoylation is limited to the Ser^2^ amino-acid residue, while Ser^3^, Ser^6^, or Ser^18^ of ghrelin 1–28 are not octanoylated. In addition, ERAT may transfer an array of long-chain natural fatty acids, not just octanoic, to as yet unknown substrates as well as to ghrelin 1–28, when the latter contains an N-terminal tripeptide extension. This specificity is reminiscent of N-myristoyltransferase. Furthermore, ERAT is a soluble enzyme, but firmly bound to the ER membrane, whereas GOAT is an insoluble integral ER membrane-spanning enzyme. If ERAT and GOAT were colocalized in the human stomach, one could envision both independent and interdependent roles of ERAT and GOAT. Although mammalian GOAT was initially considered to only add octanoic or decanoic fatty acids to Ser^3^, the laboratory of Kojima reported that GOAT effectively acylates truncated ghrelin peptides with n-hexanoic acid in cultured cells [134]. ERAT might interact by limiting in situ availability of octanoic acid to GOAT. A significant analytical point is that Ser^2^ versus Ser^3^ octanoylated ghrelin would not be distinguishable by mass spectrometry, HPLC, or probably immunologically, requiring instead mutagenesis studies, amino-acid sequencing, binding, and biological activity studies.

 Whether ERAT acylates proghrelin in vivo is not known. Albeit low in potency, Ser^2,3^ dioctanoylated ghrelin 1–28 can release GH in vivo in rats [125]. In addition, scientists at Merck laboratories demonstrated that Ser^2^ mono-octanoylated ghrelin 1–28 binds to GHS-R1a in vitro. Since octanoylated ghrelin 1–28 may exert endproduct repression of GOAT acyltransferase activity and since binding sites for GOAT, ERAT, and GHS-R1a may overlap, small amounts of Ser^2^ or Ser^2,3^ octanoylated ghrelin 1–28 might have GOAT inhibitory activity. The proposition would be that endogenous ERAT-derived ghrelin peptides may act on GOAT only at intracellular sites. 

 Notable are two ghrelin deacylation enzymes, plasma paraoxanase and gastric lysophospholipase I [156, 651]. Plasma paraoxanase is bound to high-density lipoproteins and may especially determine physiological plasma ghrelin levels. An interesting question is whether a major role of blood-borne paroxanase is to minimize the actions of octanoylated ghrelin or to maximize the actions of desacylated ghrelin. On the other hand, gastric lysophospholipase I may primarily regulate tissue ghrelin bioactivity and subsequent vagal-afferent activity as a function of the amount and type of oral fatty-acid intake. In this regard, oral octanoate increases, but oral dodecanoate decreases, plasma octanoylated ghrelin levels [134]. Accordingly, the activity of the two deacylases could be a function of amount and type of substrate as well as factors that modify synthesis of and catalysis by paroxanase and lysophospholipase I. 

 The multiple influences of the ghrelin system depend upon local and systemic posttranslational regulatory mechanisms and pleiotropic receptor-signaling in diverse target tissues [518]. Moreover, gastric ghrelin is subject to food-entrainable and circadian-clock inputs [652]. There is an additional possibility that partial overlap of ghrelin-binding properties of GHS-R1a, GOAT, and ERAT could contribute to differences and similarities between ghrelin and GHS mimetics as well as competition between full and partial GHS-R1a agonists [653]. Brown and Goldstein demonstrated that truncated ghrelin peptide analogs inhibit activity of GOAT in vitro [46]. They proposed that native octanoyl ghrelin 1–28 in higher concentrations may also inhibit GOAT. If so, one could envision that certain GHS mimetics might decrease or increase the activity of GOAT and ERAT as well as that of GHS-R1a. For example, at a low dosage Dap^3^ octanoylated ghrelin 1–28 inhibits octanoyl transferase activity in vitro indicating that GOAT is a significant site of action, whereas this analog stimulates both GH release and food intake in vivo, thus defining a GHS-R1a (ghrelin receptor) site of action [46, 654]. To assess the biochemistry, physiology, endocrinology, and therapeutic implications of peptide/non-peptide GHS mimetics and ghrelin will require combined in vitro and in vivo structure-function and dose-response analyses. 

## Figures and Tables

**Table 1:** Interactions with ghrelin.

(a) Regulation of ghrelin gene	
*Stimulation*	*Repression*
(1) GHRH (pituitary)	(1) Leptin (hypothalamus)
(2) Octanoate (stomach)	(2) Glucagon-like peptide (hypothalamus)
(3) Estradiol (stomach)	(3) Peptide YY (3-36) (hypothalamus)
(4) Glucagon (rat stomach)	(4) Insulin (stomach)
(5) Cholecystokinin (stomach)	(5) Somatostatin (pituitary, stomach)
(6) Hypoglycemia (stomach)	(6) Histamine (stomach)
(7) Acetylcholine (stomach)	(7) Hypoglycemia (brain)
(8) Leptin (stomach)	(8) Glucagon*

(b) Regulation of ghrelin receptor	

*Activation*	*Suppression*
(1) Constitutive expression	(1) Estradiol (appetitive effects)
(2) Acylghrelin	(2) GH (hypothalamus and pituitary)
(3) Estradiol (in vitro)	(3) IGF-I (pituitary)

(c) Modulation of ghrelin action	

*Potentiation*	*Inhibition*
(1) GHRH (GH release)	(1) Testosterone (dog and rat)
(2) Estradiol (GH release)	(2) Free Fatty acids (pituitary)
(3) L-arginine (GH release)	(3) Leptin (neurons)
	(3) Desacyl-ghrelin (hunger, insulinostasis)

(d) Mediation of ghrelin actions	

*Appetitive*	*Cardiovascular*
(1) NPY (↑)	(1) Nitric oxide (↑)
(2) Orexin A (↑)	(2) Extracellular-receptor activated kinases (↑)
(3) Leptin (↓)	(3) Unknown desacyl-ghrelin receptor
(4) Insulin (↓)	(4) CD36 (type B scavenger receptor)
*Stomach*	*Pituitary*
(1) GHS receptor-1a	(1) Phospholipase C
(2) ? CRH receptor-2	(2) Diacylglycerol
	(3) Protein-kinase C
	(4) Ca^2+^ signals
*Pancreas*	
(1) Inward Ca^2+^ and outward K^+^ channels	

(e) Regulation of ghrelin octanoyl-acyl transferase (GOAT)	

*Stimulation*	*Inhibition*
(1) Long-term fasting (stomach)	(1) By acylghrelin (stomach)
(2) Acetylcholine	
(3) Leptin	

* IV glucagon suppresses serum ghrelin concentrations in humans [121].

Selected References: [9, 15, 52, 88, 122–127].

**Table 2:** Experimental strategies for verifying ghrelin action.

*Genetic*
(1) transgenic silencing of ghrelin gene
(2) transgenic silencing of ghrelin receptor
(3) double knockout
(4) antisense transgene to neuronal ghrelin receptor
(5) overexpression of ghrelin
*Immunologic *
(1) immunoneutralization
(2) catalytic antibodies
*Antagonists*
(1) peptides
(2) nonpeptides
(3) RNA Spiegelmer
*Infusion of ghrelin or desacyl-ghrelin*
(1) agonism and antagonism
*Anatomic definition of ghrelin-neural network*
(1) transgenic green fluorescent protein-linked ghrelin

Selected References: [11, 65, 164–173]

**Table 3:** Reported actions of desacyl-ghrelin.

Effect of desacyl-ghrelin	Compared with acylghrelin	Reference
	shared or opposite	
Adipocytes		
(1) ↓ fat oxidation		
(2) ↑ glucose uptake		
(3) ↑ differentiation	shared	[195, 199]
(4) ↑ hypertrophy		
(5) ↓ lipolysis		
anorexigenic	opposite	[190, 196]
Antiapoptotic		
(1) islet beta cells	shared	[22, 194]
(2) cardiomyocytes		
antiinflammatory	shared and unshared	[188, 200]
body weight	opposite	[75]
cardioprotection	shared	[194, 201]
gastric motility	opposite	[196, 208]
↓ hepatic gluconeogenesis	opposite	[198]
hypotension	shared	[197]
locomotion	unknown	[209]
↑ insulin sensitivity	opposite	[210]
neurogenesis	shared	[211–214]
skeletal-muscle differentiation	shared	[193]
↓ somatic growth	opposite	[75]
vascular smooth-muscle relaxation	similar	[197]

**Table 4:** Cardiovascular actions of GHS.

↓ mean arterial pressure
↑ inotropy (myocardial tension generation)
↓ lusitrophy (tension relaxation)
↓ ventricular end-systolic pressure
↓ cardiomyocyte apoptosis
↓ endothelial apoptosis
↓ pulmonary hypertension
↑ renal perfusion
↑ coronary perfusion pressure
↑ left-ventricular ejection fraction
↓ oxygen consumption
↓ cardiac sympathetic drive

Selected References: [15, 22, 194, 197, 201].

**Table 5:** Reported modulatory messengers of ghrelin.

Messenger/mediator	Site/mechanism
(1) phospholipase C	GHS receptor-transfected cells
(2) protein kinase C	neurons
(3) cAMP potentiation	pituitary
(4) nitric oxide	vasculature
(5) urocortin-2 receptor	pancreas, stomach
(6) mitogen-activated protein kinase	cardiomyocytes
(7) extracellular-regulated kinase	cardiomyocytes
(8) potassium and calcium channels	islets, somatotropes
(9) AMP kinase	neurons, gastric mucosa

Selected References: [21, 22, 208, 238, 294, 348–352].

**Table 6:** Adipogenic effects of ghrelin.

(1) decrease fat-cell lipid export
(2) enhance lipoprotein lipase
(3) reduce insulin sensitivity
(4) stimulate preadipocyte proliferation
(5) promote adipocyte differentiation
(6) inhibit 5′-adenosine monophosphate protein kinase (AMP-kinase)
(7) augment hepatic glucose output and triacylglyceride content
(8) activate acetyl CoA carboxylase
(9) inhibit fatty acid oxidation
(10) induce leptin, sterol-response element binding protein-1c and
(11) PPAR-gamma
(12) suppress adiponectin
(13) increase appetite

Selected References: [44, 195, 198, 199, 210, 276, 293, 391–393].

**Table 7:** Key regulators of GHS-1a receptor.

Downregulation	Stimulation
glucocorticoids (rodent)	estrogen (VMN, stomach)
ghrelin (fish)	thyroxine (rat)
certain promoter hyplotypes	lactation (rat hypothalamus/pituitary)
age (human brain)	puberty (rat, pituitary)
GH (arcuate nucleus)	GHRH (pituitary)
	atherosclerosis (human)
	ghrelin-responsive corticotropinoma (human)

Selected References: [122, 187, 393, 421–425].

**Table 8:** Diabetogenic and antidiabetogenic actions of ghrelin.

Prodiabetic effects	Antidiabetic effects
stimulation of hepatic glucose output	chronic ↑ GH (lipolysis)
adipogenesis*	increase lean-body mass (chronic)
inhibition of insulin secretion	decrease oxygen consumption
appetite enhancement	increase uncoupling protein-1
acute free-fatty acid release* (human)	
Antithermogenesis	
decreased sympathetic outflow	

*reduces tissue insulin action.

See Tables 3 and 6 for selected references.

**Table 9:** Issues concerning longterm administration of ghrelin antagonists.

*Desirable*
↓ hyperglycemia
↑ insulin secretion
↓ appetite and food intake
↑ fat oxidation
↑ LH secretion?

*Undesirable*
↓ gastric emptying
↑ blood pressure (vasoconstrict)
↑ cardiac oxygen consumption?
↓ neoplastic apoptosis?
↑ inflammatory mediators?
↓ bone growth?
↑ gastric alkalinity and mucosal permeability?
↓ GH secretion (female)?
↓ neurogenesis (brainstem, cortex)?
↑ hypoglycemia during prolonged fast?
